# Editorial

**DOI:** 10.34763/jmotherandchild.20202402si.2028.000001

**Published:** 2020-11-10

**Authors:** Stephanie Grunewald

## Why rare disorders matter: lessons to learn from Inborn Errors of Metabolism

As the journal *Developmental Period Medicine/Medycyna Wieku Rozwojowego* has recently taken off as *Journal of Mother and Child (JMC)/Medycyna Wieku Rozwojowego*, this is its first special issue dedicated to Inborn Errors of Metabolism (IEM).

It brings together papers on several areas of IEM, reflecting on the widely ranged aspects of the care for patients with IEM.

The majority of IEM are rare diseases caused by a single gene defect, encoding for enzymes that facilitate the conversion of substrates. This often leads to accumulation of toxic substances or deficiencies of essential metabolites.

By definition, a rare disorder affects a small number of people, 1:2,000 (according to the definition used in European countries) and even lower – so why bother?

First of all, the cumulative impact of those – and there are close to 8,000 known IEM – is significant and patients deserve early diagnosis and treatment. So sharing the experience of the diagnosis and prognosis, management and treatment of IEM in this issue is highly appreciated.

With improved diagnostic tools and early diagnosis being facilitated in some IEM by newborn screening and increasing favourable therapeutic options, patients are surviving longer and with that the natural history evolves further. Detailed examples are discussed in this special issue, e.g. by Davison, focusing on Pompe disease, and by Forny et al., reviewing the expanding spectrum of long-term complications in isolated methylmalonic aciduria. Prof Jaeken’s review on selected subtypes of congenital disorder of glycosylation (CDG) gives further insight on unusual presentation, different phenotypes and a novel biochemical/genetic mechanism and discusses some treatment.

Stepien reviews in detail hormonal dysfunctions in adults with IEM, whereas Haeberle and Huemer discuss the diagnostic pathways of patients presenting with a key biochemical hallmark such as hyperammonaemia or elevated homocysteine levels to unreveal the underlying metabolic condition.

And then, research into these rare conditions has had a major impact in discovering pathomechanisms that can help to understand the researched metabolic condition but might also increase the understanding of non-metabolic diagnosis. Sperl et al. not only give some very interesting examples of unrevealing mitochondrial disorders, but also guide us through the changing diagnostic pathways and tools used in diagnosing patients with mitochondriopathies over time.

And if that was not enough to justify a special issue on IEM:

Understanding the very detail of metabolic pathomechanism, Prof Peter Clayton (1), one of the most eminent experts in the field of IEM, contributed lately a fascinating discussion on a possible link of IEM and the current most horrendous global health treat – COVID-19: in a very recently published paper, he questions that the susceptibility to severe COVID-19 disease might be due to an underlying IEM. This is on the background of the findings of genome-wide association analysis of those SARS-CoV-2 patients in Italy and Spain who developed the severe respiratory complications. Ellinghaus et al. (2) detected some statistical relevant cross-replication associations. Among six genes at chromosome 3p21.21, one gene, *SLC6A20*, encodes for a proline transporter. Prof Clayton provides a fascinating possible link of altered proline concentrations interfering with protein folding or directly impacting on immune response. To proof this, he recommends the application of standard metabolic testing, readily available, measuring and comparing the proline content in individuals with favourable and poor outcome. Should proline play a key link to the susceptibility to severe COVID-19, there might even be a therapeutic role to be discussed.

Innovative research methods for studying new therapies in IEM are essential and have been successfully implemented in this ever-growing field. As the prevalence of rare conditions might be 1:2,000 and even lower, clinical research on treatment for IEM is challenging. Evolving strategies like gene therapy is of particular interest and is discussed in this issue by Yilmaz et al.

Not only are individual IEM complex in themselves, any patient has his/her individual characteristics that need to be taken into account when providing care to these patients. Burgard advocates for a holistic approach as a joint project of the clinical team and the patient. This approach would easily be adjustable for any non-metabolic patient.

There are many lessons in life to be learned – without doubt, the field of IEM is probably one of the most resourceful areas in medicine to share its lessons learned with the broader medical community helping unrevealing new pathways and pathomechanisms.
